# A quantitative study on the formation of *Pseudomonas aeruginosa* biofilm

**DOI:** 10.1186/s40064-015-1029-0

**Published:** 2015-07-28

**Authors:** Ashwin Kannan, Pennathur Gautam

**Affiliations:** Visvesvaraya National Institute of Technology, Nagpur, 440 010 India; Centre for Biotechnology, Anna University, Chennai, 600 025 India

**Keywords:** *Pseudomonas aeruginosa*, Biofilm, Atomic force microscopy, Adhesion force, Extracellular polymeric material, Temperature

## Abstract

Biofilms are bacterial cells in a matrix of extracellular polymeric substance. The formation of biofilm depends on the microenvironment. In this study, the effect of temperature on *Pseudomonas aeruginosa* biofilm formation was evaluated with respect to three parameters—the mass of biofilm formed, the production of extracellular polysaccharide and the adhesion force. The results indicate that biofilm biomass (2.8, A590), extracellular polysaccharide production (1240 ± 40 µg) and adhesion force (10.8 ± 0.2 nN) were highest at 37°C. The results also suggest that biofilms formed at 37°C would have a higher mechanical stability (than biofilms grown at 28, 33 and 42°C).

**Electronic supplementary material:**

The online version of this article (doi:10.1186/s40064-015-1029-0) contains supplementary material, which is available to authorized users.

## Background

Power plants and large scale industries are located near water bodies because of their need for large amounts of water (for use as coolant). The discharge from soap industries results in the accumulation of sodium palmitate (main constituent of soaps) in these water bodies. When the magnesium ions (present in water bodies) react with sodium palmitate, magnesium palmitate is formed. Bacterial cells use magnesium palmitate as a carbon source for their growth and development (Ashwin et al. 2014). This results in the formation of biofilms since microorganisms exist predominantly as biofilms in water bodies (Costerton et al. 1995). The discharge (of water used as coolant) raises the temperature of the water body in the immediate vicinity of the mixing zone, thereby forming a temperature gradient in the water body (Langford 1990). The formation of a temperature gradient imparts a thermal stress on the bacterial cells, which ultimately influences the formation of biofilm.

Hostacka et al. (2010) reported the effect of temperature on formation of *Pseudomonas aeruginosa* biofilm by estimating the biomass of biofilm formed. Recently, Zhou et al. (2014) reported the effect of temperature on formation of *P. aeruginosa* biofilm by estimating the concentration of polysaccharide and protein concentration in biofilm. However a study on the effect of temperature on adhesion force and surface topography of *P. aeruginosa* biofilm is lacking. In this study, the effect of temperature on *P. aeruginosa* biofilm formation was evaluated with respect to three parameters—the mass of biofilm formed, the production of extracellular polysaccharide and the adhesion force. In addition, surface topography of biofilm was also evaluated.

## Methods

The organism *P. aeruginosa* (MTCC 2297) was chosen since it is a model organism used in biofilm research. *P. aeruginosa* grows over a wide range of temperatures. The media for formation of biofilm consisted of M9 minimal media with 1% (w/w) magnesium palmitate as the carbon source (Ashwin et al. 2014). Magnesium palmitate was prepared according to the procedure given by Quraishi et al. (2012). Equimolar concentration of palmitic acid and potassium hydroxide were mixed and the suspension was heated continuously to yield potassium palmitate. Magnesium chloride, in slight excess of what was required for the stoichiometric reaction, was added to the solution. The product was then cooled and the precipitate was filtered and washed with water and acetone. Magnesium palmitate thus obtained was dried to constant weight.

The samples for inductively coupled plasma (ICP) analysis (to determine the concentration of metal ions) were prepared according to the protocol given by Cruz et al. (2011). The biofilm sample was centrifuged at 12,500*g* for 15 min and then acid digested in concentrated nitric acid for 1 h. The supernatant (of the digested sample) was diluted using double distilled water and the metal concentration was determined using ICP optical emission spectrometry (Perkin Elmer Optima 5300 DV).

Biofilm biomass was estimated using the protocol of Merritt et al. (2011). Biofilm samples were prepared according to the procedure given earlier (Ashwin et al. 2014). Biofilm samples were incubated in 0.1% crystal violet solution for 10 min. The samples were then rinsed with water and the bound crystal violet was extracted using 95% crystal violet. A590 of the solubilizing buffer was then measured.

The extracellular polysaccharide produced was quantified using the procedure given by Forde and Fitzgerald (1999) and Parkar et al. (2001). The cells were centrifuged at 12,500*g* for 15 min and were then resuspended in 5 ml of sodium hydroxide. The solution was heated for 15 min. The cells were then removed by centrifuging at 12,500*g* for 30 min. 99.9% (v/v) ethanol was added to the supernatant and incubated at 40°C to precipitate the polysaccharides. The precipitated polysaccharides were then dissolved again in 10 ml of sterile water. To 1 ml of this sample, 8 ml of 98% (v/v) sulphuric acid was added and the mixture was cooled for 10 min. 1 ml of 1% (w/v) of cold tryptophan was added to this mixture and heated for 15 min to effect hydrolysis. The amount of polysaccharide was then estimated by measuring A500. AFM images of biofilm were resolved in air using JPK Nanowizard II. The images were acquired in contact mode using Mikromasch CSC38 probes (Additional file 1: Figure S1). The spring constant of the cantilevers used were 0.03 N/m (Ashwin et al. 2014). A total of eight force-distance curves (on each biofilm sample) were generated to evaluate the biofilm adhesion force. A typical force curve is given (Additional file 2: Figure S2). All experiments were performed in triplicate.

## Results and discussion

The effect of temperature on *P. aeruginosa* biofilm formation was evaluated with respect to three parameters—the mass of biofilm formed, the production of extracellular polysaccharide and the adhesion force. Quantification of biofilm biomass was done using the dye crystal violet (Merritt et al. 2011). Biofilm biomass formed at temperatures 28, 33, 37 and 42°C were 1.25 (O.D), 2.20 (O.D) 2.80 (O.D) and 2.30 (O.D) respectively (Table 1). The results show that biofilm biomass was highest for biofilm sample formed at 37°C. This is due to increased production of extracellular matrix material by *P. aeruginosa* cells at 37°C.

**Table 1:** Biofilm formation evaluated at different growth temperature supporting information

Growth temperature (°C)	Adhesion force (nN)	Surface roughness R_a_ (nm)	Biofilm biomass (O.D)	Extracellular polysaccharide (µg)	Concentration of metal ions in biofilm (mg/l)
42	6.6 ± 0.1	80	2.30 ± 0.1	1240 ± 40	Ca, 0.055 Mg, 1.58
37	10.8 ± 0.2	139	2.80 ± 0.1	1570 ± 57	Ca, 0.10 Mg, 2.36
33	5.4 ± 0.2	118	2.20 ± 0.1	1210 ± 35	Ca, 0.046 Mg, 1.42
28	4.7 ± 0.1	185	1.25 ± 0.1		Ca, 0.03 Mg, 0.78

Adhesion force of biofilm is a measure of adhesiveness of biofilm (probe-biofilm interaction force). Adhesion force of biofilm grown at 28, 33, 37 and 42°C were 4.7, 5.4, 10.8 and 6.6 nN respectively (Table 1).

The results indicate that biofilm adhesion force was highest for biofilm sample formed at 37°C. This is due to increased production (and accumulation) of extracellular matrix material by *P. aeruginosa* cells at 37°C (Ashwin et al. 2014; Oh et al. 2007, 2009; Fang et al. 2000). The increased production of extracellular polymeric material is due to higher metabolic rate of *P. aeruginosa* cells at 37°C.

Extracellular polysaccharides play an important role in biofilm formation since it is only when extracellular polysaccharides are produced that cell aggregation and biofilm formation occur. This is because the extracellular polysaccharides act as ‘cement’ in holding the cells together (Sutherland 2001).The amount of extracellular polysaccharide produced by *P. aeruginosa* cells grown at 28, 33, 37 and 42°C were 890, 1,210, 1,570 and 1,240 µg respectively (Table 1). The results show that extracellular polysaccharide production was highest for biofilm sample formed at 37°C.

The higher amount of calcium and magnesium ions incorporated in the biofilm (formed at 37°C: Table 1) suggests that biofilm (formed at 37°C) would have a higher mechanical stability (than biofilms grown at 28, 33 and 42°C). This is because calcium and magnesium ions have been reported to bind with negatively charged polysaccharides and have also been implicated in cell–cell binding mechanisms (Korstgens et al. 2001; Lattner et al. 2003).

Surface topography of biofilm was characterized by evaluating its surface roughness. Surface roughness of biofilm is an important parameter that is characteristic of growth conditions and has been reported to have an effect on the rate of diffusion of nutrients, thickness of dissolved oxygen boundary layer and mass transfer resistance (Zhang et al. 1994). Surface roughness of biofilm was evaluated using parameter R_a_. This parameter measures the arithmetic average of deviation about the mean profile line (Ashwin et al. 2014). Table 1 also shows that biofilm sample grown at 28°C had the highest surface roughness, although no specific trend was observed. This indicates that growth temperature has a significant effect on the surface topography of *P. aeruginosa* biofilm.

The aforementioned results show that *P. aeruginosa* biofilm (strain MTCC 2297) formed at 37°C produce higher amount of extracellular polysaccharide and also have a higher biomass, higher adhesion force and greater mechanical stability than biofilms formed at other temperatures (in the temperature range 28–42°C).

## Conclusion

The effect of temperature on *P. aeruginosa* biofilm formation was evaluated with respect to three parameters—the mass of biofilm formed, the production of extracellular polysaccharide and the adhesion force. The results indicate that biofilm biomass (2.8, A590), extracellular polysaccharide production (1240 ± 40 µg) and adhesion force (10.8 ± 0.2 nN) were highest at 37°C. The results also suggest that biofilms formed at 37°C would have a higher mechanical stability (than biofilms grown at 28, 33 and 42°C). The results indicate that temperature has a significant effect on formation of *P. aeruginosa* biofilm.

## Additional files

Additional file 1:
**Figure S1.** AFM (Vertical Deflection) images of biofilm formed at different temperatures: A- 28°C. B- 33°C. C- 37°C. D- 42°C. Additional file 2:
**Figure S2.** A typical force curve.
