# Supplementary figures and images for: A quantitative study on the formation of Pseudomonas aeruginosa biofilm
Source: Springerplus. 2015 Jul 28;4:379. doi: 10.1186/s40064-015-1029-0 (PMC4516152; doi:10.1186/s40064-015-1029-0)

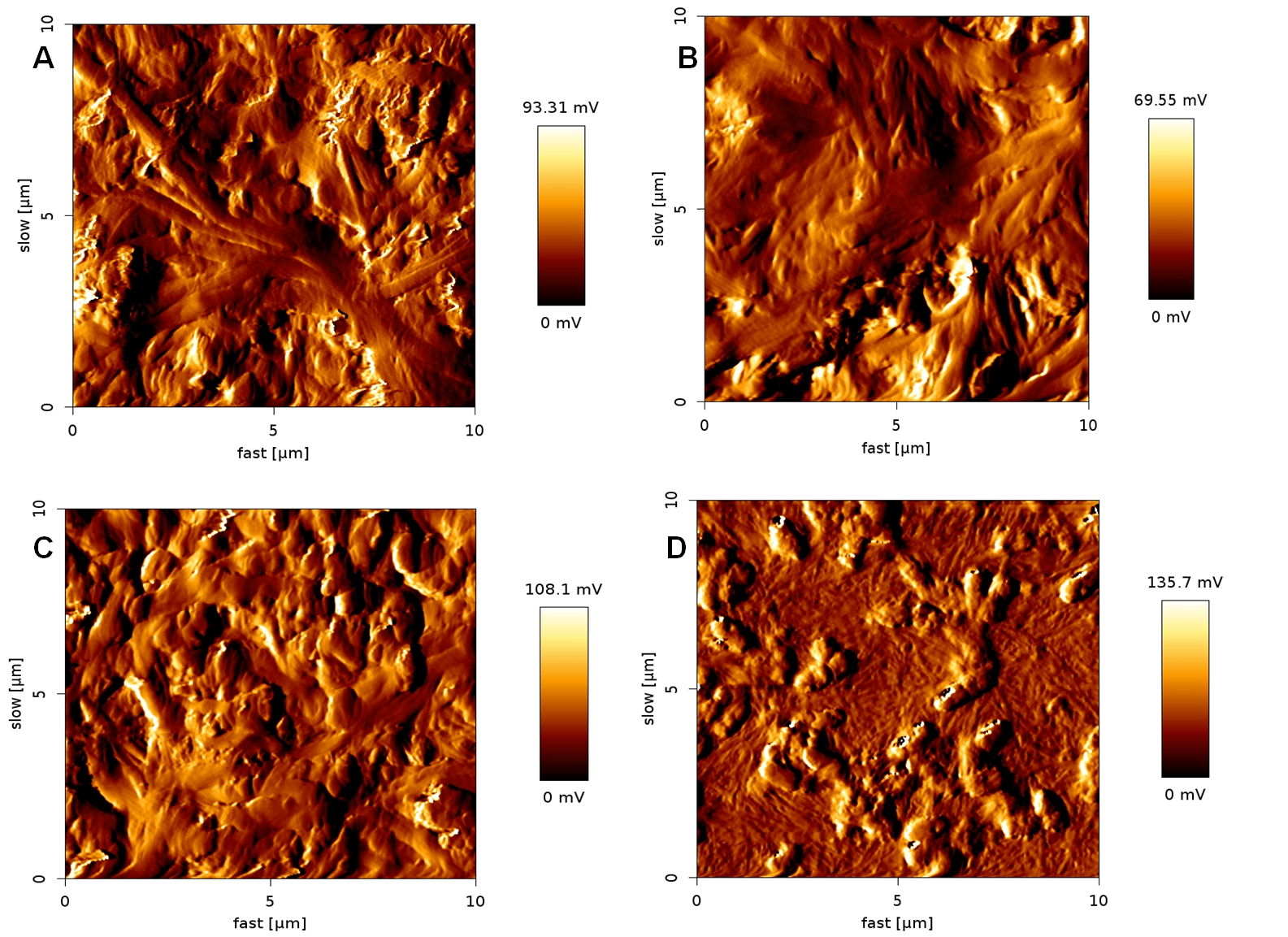

Supplement: Additional file 1: — Figure S1. AFM (Vertical Deflection) images of biofilm formed at different temperatures: A- 28°C. B- 33°C. C- 37°C. D- 42°C. [file 40064_2015_1029_MOESM1_ESM.tiff]

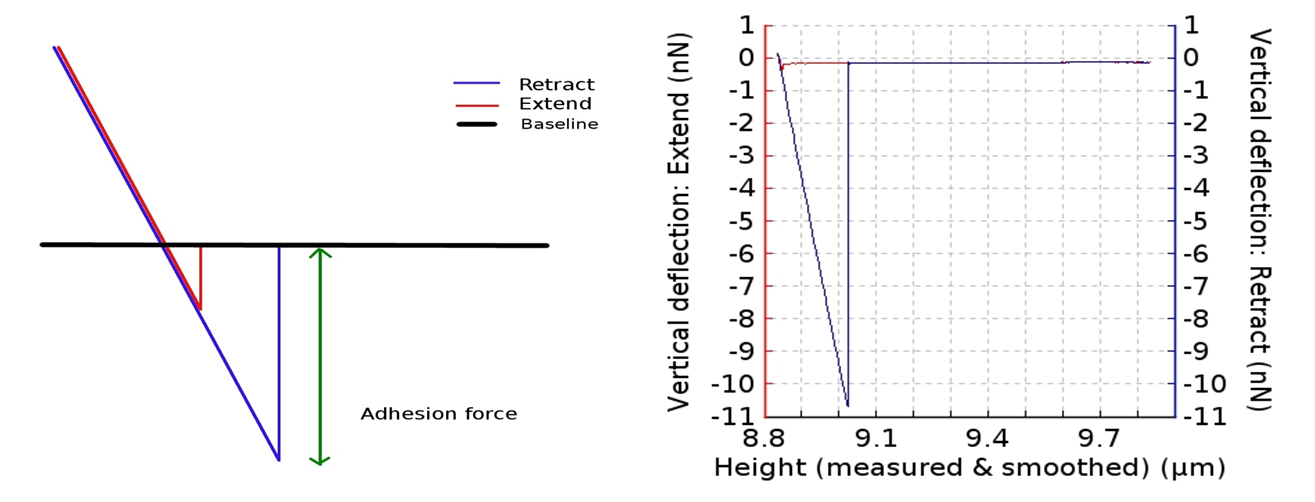

Supplement: Additional file 2: — Figure S2. A typical force curve. [file 40064_2015_1029_MOESM2_ESM.tiff]
